# Early and mid-term results of transapical and right axillary artery cannulation for acute aortic dissection

**DOI:** 10.1186/1749-8090-8-S1-O42

**Published:** 2013-09-11

**Authors:** T Terasaki, T Takano, T Fujii, K Komatsu, Y Ohtsu, Y Wada, T Seto, J Amano

**Affiliations:** 1 Department of Cardiovascular Surgery, Shinshu University School of Medicine, Matsumoto, Japan

## Background

We combined transapical cannulation (TAC) and right axillary artery cannulation (RAAC) for the repair of AAAD to reduce mortality and morbidity when malperfusion risk existed. We evaluated early and mid-term outcomes of AAAD repair with TAC and RAAC.

## Methods

Between October 2009 and March 2012, 23 patients of AAAD received graft replacement with TAC and RAAC combination. Patients’ age was 54.3±13.5 years old. Preoperative malperfusion was presented in 16 patients (69.6%). CPB was initially started with RAAC through the right armpit and right atrial drainage, and then TAC was applied through apex. We retrospectively investigated mortality and morbidity, and cardiac functions by echocardiography during hospital stay and once a year after operation.

## Results

Total arch replacement was performed in all patients. In-hospital mortality was 4.3%, and no patient developed intraoperative malperfusion. Intraoperative stroke occurred in 1 patient (4.3%), and 3 patients (13.0%) suffered from delayed stroke (10-24 days). These delayed strokes might be caused by cardiogenic thrombus, although no intracardiac thrombus was found. Mean ejection fraction was 66.1±10.9 % in early periods and 73.1±8.7% mid-term periods. There was no LV asynergy or intra-cardiac thrombus in echocardiography of both studies of early and mid-term period.

## Conclusions

TAC with RAAC is a safe and effective procedure to reduce the risk of operation in acute type A aortic dissection. TAC may not affect cardiac functions although it might have potential risk of delayed stroke.

